# Spurious Thrombocytosis in the Setting of Hemolytic Anemia and Microcytosis Secondary to Extensive Burn Injury

**DOI:** 10.4274/tjh.2017.0466

**Published:** 2018-08-05

**Authors:** Mohammad Faizan Zahid, Mohamed S. Alsammak

**Affiliations:** 1 Temple University Hospital, Clinic of Internal Medicine, Philadelphia, Pennsylvania, USA; 2 Temple University Hospital, Clinic of Pathology and Laboratory Medicine, Philadelphia, Pennsylvania, USA

**Keywords:** Spurious, Thrombocytosis, Burn, Platelets, Microcytosis

## To the Editor,

A 57-year-old man was brought to our emergency department from a house fire. On physical examination, he was nonresponsive, hypotensive, and tachycardic with full-thickness skin burns covering the entirety of the body except the lower back (>98% of his body surface area). He was intubated and aggressively resuscitated with IV fluids and multiple pressors for circulatory support.

**Figure 1:**
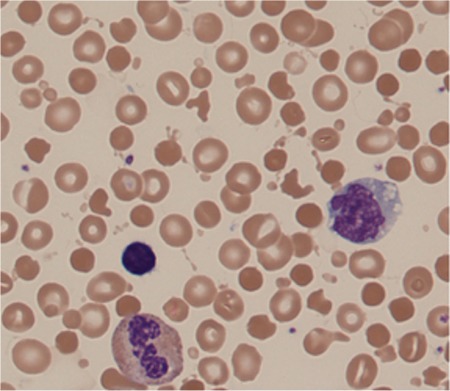
Widespread red blood cell fragmentation, budding, spherocytes, and microspherocytes were revealed by microscopic examination.

A complete blood count (CBC) showed normal hemoglobin (14.5 g/dL) with leukocytosis (23.6x10^9^/L) and thrombocytosis (979x10^9^/L). Repeat CBCs also showed thrombocytosis (815x10^9^/L and 1121x10^9^/L). Microscopic examination of the peripheral blood smear showed widespread red blood cell (RBC) fragmentation, budding, spherocytes, and microspherocytes (Figure 1). Manual platelet count estimates on the peripheral blood smear demonstrated a count of 173x10^9^/L. The patient remained in intractable hypotension and eventually went into cardiac arrest.

The aforementioned findings are seen in patients with severe burns due to direct thermal injury of RBCs circulating through the skin. Exposure to extreme heat leads to the denaturation of RBC membrane proteins, which results in hemolysis, RBC fragmentation, and vesiculation [1]. The loss of cell membrane causes the RBCs to lose their biconcavity and assume the shape of spherocytes and microspherocytes [1]. These RBC fragments and microspherocytes persist in the peripheral circulation for several days until completely removed from circulation by the reticuloendothelial system in the spleen. They are counted as platelets by aperture-based automated analyzers due to their size, leading to falsely elevated platelet counts in cases of acute burns [1,2]. Although reactive thrombocytosis can be seen in acute injury as recently reported by Sapanara et al. [2] in a similar burn case of a 48-year-old woman, such instances should always prompt a microscopic examination of the peripheral smear to confirm if in fact the platelet count is elevated. A manual count of platelets on peripheral smear from that patient (as in our case) revealed a normal platelet count. Such examples emphasize the importance of correlating the peripheral smear with automated CBC results.
